# Reversal of Cadmium-induced Oxidative Stress in Chicken by Herbal Adaptogens *Withania Somnifera* and *Ocimum Sanctum*

**DOI:** 10.4103/0971-6580.72671

**Published:** 2010

**Authors:** K. Bharavi, A. Gopala Reddy, G. S. Rao, A. Rajasekhara Reddy, S. V. Rama Rao

**Affiliations:** Department of Pharmacology and Toxicology, NTR College of Veterinary Science, Gannavaram, Krishna (District), Hyderabad - 30, Andhra Pradesh, India; 1 Department of Pharmacology and Toxicology, College of Veterinary Science, Rajendranagar, Hyderabad - 30, Andhra Pradesh, India; 2 Department of Poultry Science, College of Veterinary Science, Rajendranagar, Hyderabad - 30, Andhra Pradesh, India; 3 Project Directorate on Poultry, Hyderabad - 30, Andhra Pradesh, India

**Keywords:** Adaptogens, cadmium toxicity, chicken, *Ocimum sanctum*, *Withania somnifera*

## Abstract

The present study was carried out to evaluate the herbal adaptogens *Withania somnifera* and *Ocimum sanctum* on cadmium-induced oxidative toxicity in broiler chicken. Cadmium administration at the rate of 100 ppm orally along with feed up to 28 days produced peroxidative damage, as indicated by increase in TBARS, reduction in glutathione (GSH) concentration in liver and kidney, and increase in catalase (CAT) and superoxide dismutase (SOD) of erythrocytes. Herbal adaptogens *Withania somnifera* roots and *Ocimum sanctum* leaf powder administration at the rate of 0.1% through feed reversed the antioxidant enzyme of RBC, i.e., CAT and SOD, nonenzymatic antioxidants GSH and lipid peroxidation marker TBARS of liver and kidney. Liver and kidney tissue repair and normal function was assessed by alanine aminotransaminase for liver and creatinine and blood urea nitrogen for kidney. In conclusion, oral administration of *Withania somnifera* root and *Ocimum sanctum* leaf powder prevented cadmium-induced peroxidation of tissues.

## INTRODUCTION

Cadmium, a toxic heavy metal, is being released in very large amounts into the environment by anthropogenic activity. It is reported that increased concentrations of cadmium in agricultural soil are known to come from application of phosphate fertilizer, sewage sludge, waste water, and pesticides.[1] Furthermore, it is also established that mining activities, smelting of metalliferous ores with high cadmium content and industrial application of cadmium in pigments, plastic stabilizers, and nickel cadmium batteries may result in widespread agricultural pollution.[2] Cadmium is water soluble and is easily transferred efficiently from soil to plants that may affect target species, if there is intake of feed ingredients from a contaminated plant source.[3] Cadmium present in soil can be absorbed by plants and accumulate in it. Poultry is highly susceptible to cadmium toxicity, because cadmium intoxication may occur through feed ingredients of plant origin and also from dicalcium phosphate, fish meal, and shell grid.

Accumulation of cadmium in tissues may lead to decreased rate of growth. Akyolcu *et al*.[4] observed that cadmium administration resulted lower body weights and higher tissue cadmium concentrations. Uyanik *et al*.[5] also reported that cadmium administration at the rate of 100 ppm resulted in suppression of live weight, alteration in biochemical parameters, damage in kidney, liver and bursa fabricius, and accumulation in liver, kidney, and muscle tissues. The immediate consequence of exposure to cadmium *in vivo* is stimulation of reactive oxygen species (ROS) production in the mitochondrial electron transfer chain, inhibition of NADPH oxidase activity in the plasma,[6] and depletion of physiological antioxidants like reduced glutathione (GSH).[7] By increasing the production of free radicals, cadmium produces oxidative stress which has been proposed as a mechanism for cadmium toxicity in a number of tissues such as kidney[8] and liver,[9] the primary target of Cd toxicity. The increased ROS cause lipid peroxidation, DNA damage, depletion of sulphydryls, and altered calcium homeostasis.[10]

Oxidative damage by free radicals can be prevented by the use of antioxidants such as Vit E, β-carotene and coenzyme Q[11] and second line inside the cell, water soluble antioxidant scavengers, vitamin C, GSH peroxidase, superoxide dismutase (SOD), and catalase (CAT). *Withania somnifera*, popularly known as ashwagandha, is widely considered as the Indian ginseng and it promotes physical and mental health, rejuvenate the body in debilitated conditions, and increase longevity.[12] Mishra *et al*.[13] reported better response of *Withania somnifera* administration on growth, feed consumption, efficiency of feed conversion, and decreased mortality rate in broiler chicks, and improvement in antioxidant status and reduction in lipid peroxidation (LPO) in mice.[14] In traditional system of medicine, different parts of *Ocimum sanctum* have been recommended for the treatment of bronchitis, asthma, malaria, diarrhea, chronic fever, etc. It has also been suggested to possess antifertility, anticancer, antidiabetic, antispasmodic, analgesic, and adaptogenic actions.[15] Monica and Gupta[16] reported that *Ocimum sanctum* leaf powder supplement in feed increased the live weight gain and immunity in broiler chicken. *Withania somnifera* and *Ocimum sanctum* are most often described as adaptogens.[17]

By keeping the above facts in view, an experimental study was planned on broilers to assess the mechanisms, antioxidants disturbance, and toxicity of cadmium and amelioration by herbal adaptogens such as *Withania somnifera* and *Ocimum sanctum*.

## MATERIALS AND METHODS

### Chicken

A total of 60 male broiler chicks (Cobb strains) of a-day-old age were procured from the Venkateshwara Hatcheries, Hyderabad, AP, India, and reared in a battery brooder. They were fed with standard basal diet throughout their life. Birds of all groups were vaccinated with new castle disease vaccine on 7^th^ and 28^th^ day and infectious bursal disease vaccine on 10^th^ day. Weekly body weights of all birds were recorded from the day of hatch till the completion of experiment. Before commencing the work, permission from institutional animal ethics committee was obtained.

### Experimental design

A total of 60 male broiler chicks (Cobb strain) were randomly divided into four groups consisting of 15 in each group. All the birds were provided with respective feed and water ad libitum throughout the experiment. The treatment schedules of various groups of birds are as follows: Group 1 birds were fed with basal diet throughout the experiment (1 – 42 days). Group 2 to 4 birds were fed with basal diet mixed with 100 ppm cadmium as cadmium chloride up to 28 days (4 weeks) and from 29^th^ day onwards, Group 2 birds were fed with normal basal diet alone. Group 3 birds were fed with basal diet mixed with 0.1% root powder of *Withania somnifera* and Group 4 birds were fed with basal diet mixed with 0.1% leaf powder of *Ocimum sanctum*. The dose of 100 ppm of cadmium as cadmium chloride to induce oxidative toxicity was selected as per Uyanik *et al*.[5] who reported that 100 mg/kg of cadmium significantly altered the performance, biochemical parameters, and antioxidant parameters.

### Blood and tissue collection from birds

Blood samples were collected from wing veins on 28^th^ and 42^nd^ day from all the birds with anticoagulant (Alsever’s solution) in each group for assay of SOD and CAT of erythrocytes, and without anticoagulant to separate serum immediately and aliquoted for assays. Birds were sacrificed by cervical dislocation at the end of sixth week that is on the same day of blood collection, and liver and kidney were collected. Kidney and liver after excision were washed thoroughly with ice cold saline (0.9%) and prepared in to 10% homogenate with 0.2M Tris HCl buffer (pH 7.2). Cytosolic sample of liver and kidney homogenate was obtained by centrifuging at 10,000 for 30 min at 4°C.

### Biochemical analysis

CAT activity in fresh blood (erythrocytes) was estimated by Caliborne[18] and SOD activity by Marklund and Marklund[19] method. GSH content in liver and kidney was was determined by Moron *et al*.[20] method, and Levels of thiobarbituric acid reactive substance (TBARS), which is LPO marker, in liver and kidney were assessed by following Subramanian *et al*.[21] method. Alanine aminotransferase was analyzed by the method of Bergmeyer *et al*.,[22] creatinine was estimated by Apple *et al*. method,[23] and blood urea nitrogen (BUN) was estimated by method prescribed by Wybenga *et al*.[24] Total protein in liver and kidney homogenate was quantified by the procedure of Lowry *et al*,[25] using bovine serum albumin as the standard. The data were subjected to statistical analysis by applying one way ANOVA using Statistical Package for Social Sciences (SPSS) 10^th^ version. Differences between means tested using Duncan’s multiple comparison test and significance was set at *P*<0.05.

## RESULTS

### Body weight gain

Groups treated with cadmium showed significant reduction in body weights at the end of fourth week. Following treatment with herbal adaptogens from fourth to sixth week in groups 3 and 4, the body weights increased compared with cadmium control group 2 [Table 1].

**Table 1:** Effect of herbal adaptogens *Withania somnifera* and *Ocimum sanctum* on cadmium-induced alteration of average body weight, erythrocyte antioxidant enzymes, and liver and kidney function biomarkers

Parameter		Group 1	Group 2	Group 3	Group 4
Performance parameter					
Average body weight	4^th^ week	949.7 ± 13.9^cA^	592.8 ± 14.08^bA^	574.2 ± 20.72^aA^	560.4 ± 13.00^aA^
	6^th^ week	1551.9 ± 20.02^cB^	824.41 ± 10.76^aB^	1219.1 ± 25.56^bB^	1248.33 ± 31.05^bB^
Erythrocyte antioxidant enzymes					
SOD	4^th^ week	42.74 ± 0.42^aA^	82.68 ± 0.58^bA^	82.55 ± 0.61^bA^	82.70 ± 0.56^bA^
	6^th^ week	45.96 ± 0.73^aB^	91.43 ± 0.65^cB^	56.56 ± 0.30^bB^	66.09 ± 0.39^bB^
CAT	4^th^ week	2.55 ± 0.02^aA^	5.89 ± 0.01^bA^	5.90 ± 0.02^bA^	5.92 ± 0.07^bA^
	6^th^ week	3.17 ± 0.01^aB^	6.63 ± 0.16^cB^	4.03 ± 0.01^bB^	4.49 ± 0.005^bB^
Liver and kidney function biomarkers					
ALT	4^th^ week	16.75 ± 0.83^aA^	56.42 ± 3.41^bA^	54.60 ± 3.97^bA^	57.61 ± 4.05^bA^
	6^th^ week	16.44 ± 0.69^aA^	58.76 ± 1.88^cA^	28.33 ± 4.36^bB^	27.63 ± 0.65^bB^
BUN	4^th^ week	4.00 ± 0.03^aA^	7.46 ± 0.18^bA^	7.47 ± 0.11^bA^	7.42 ± 0.17^bA^
	6^th^ week	6.58 ± 0.84^aB^	9.55 ± 0.17^cB^	7.99 ± 0.25^bB^	8.31 ± 0.24^bB^
Creatinine	4^th^ week	0.50 ± 0.02^aA^	0.69 ± 0.02^bA^	0.69 ± 0.03^bA^	0.72 ± 0.01^bA^
	6^th^ week	0.60 ± 0.01^aB^	1.28 ± 0.01^cB^	0.85 ± 0.009^bB^	0.94 ± 0.01^bB^

Values are mean ± SEM (n = 15) one-way ANOVA. Means with different alphabets as superscripts differ significantly (*P*<0.05); capital alphabets (vertical comparison); small alphabets (horizontal comparison). Group 1: 1 to 42 days - Basal diet control. Group 2: 1 to 28 days - Basal diet mixed with 100 ppm cadmium 29 to 42 days - Basal diet only. Group 3: 1 to 28 days - Basal diet mixed with 100 ppm cadmium, 29 to 42 days - Basal diet mixed with 0.1% root powder of *Withania somnifera*. Group 4: 1 to 28 days - Basal diet mixed with 100 ppm cadmium, 29 to 42 days - Basal diet mixed with 0.1% leaf powder of *Ocimum sanctum*. SOD - superoxide dismutase; CAT - catalase; ALT - alanine aminotransaminase; BUN - blood urea nitrogen; SEM - standard error mean

### Blood antioxidant enzymes

Cadmium-treated groups showed significant higher (*P*<0.05) values of erythrocyte super oxide dismutase and CAT compared with the control group 1 at the end of fourth week. In groups 3 and 4 treated with herbal adaptogens from fourth to sixth week, the values were significantly reduced [Table 1].

### Nonenzymatic antioxidants and lipid peroxidation of liver and kidney

The cadmium control toxic group 2 exhibited significant decrease in GSH and increase in TBARS concentration of liver and kidney tissues when compared with basal diet control group. In contrast, herbal adaptogens treatment in groups 3 and 4 showed significant increase in the liver and kidney tissue GSH concentration and decrease in the TBARS concentration [Table 2].

**Table 2:** Effect of herbal adaptogens *Withania somnifera* and *Ocimum sanctum* on cadmium induced tissue lipid peroxidation and GSH levels of liver and kidney

Groups	TBARS		GSH	
	Liver	Kidney	Liver	Kidney
Group 1	141.93 ± 3.48^a^	150.92 ± 7.85^a^	72.41 ± 2.83^e^	75.58 ± 1.45^e^
Group 2	180.99 ± 4.17b	236.10 ± 18.64^c^	31.96 ± 2.09^a^	37.65 ± 0.71^a^
Group 3	148.18 ± 8.25^a^	163.75 ± 5.23^ab^	63.75 ± 6.53d^de^	68.16 ± 1.53d
Group 4	151.12 ± 4.99^a^	163.30 ± 7.86^ab^	63.73 ± 2.27d^de^	69.98 ± 1.68d^de^

Values are mean ± SEM (n = 15) one-way ANOVA. Means with different alphabets as superscripts differ significantly (*P*<0.05); small alphabets (vertical comparison).Group 1: 1 to 42 days - Basal diet control. Group 2: 1 to 28 days - Basal diet mixed with 100 ppm cadmium 29 to 42 days - Basal diet only. Group 3: 1 to 28 days - Basal diet mixed with 100 ppm cadmium 29 to 42 days - Basal diet mixed with 0.1% root powder of *Withania somnifera*. Group 4: 1 to 28 days - Basal diet mixed with 100 ppm cadmium 29 to 42 days - Basal diet mixed with 0.1% leaf powder of *Ocimum sanctum*. TBARS - thiobarbituric acid reactive substance; GSH – glutathione; SEM - standard error mean

### Liver and kidney toxicity marker

At the end of fourth week, alanine aminotransaminase (ALT) concentration was significantly increased in cadmium-treated groups 2 to 4 and following treatment with herbal adaptogens in groups 3 and 4 from fourth to sixth week, there was significant decrease in ALT activity compared with cadmium control group 2 at the end of sixth week [Table 1]. At the end of fourth week, BUN and serum creatinine concentration was significantly increased in cadmium-treated groups 2 to 6. However, following treatment with adaptogen plants from fourth to sixth week, there was significant decrease in BUN and serum creatinine levels in groups 3 and 4 at the end of sixth week [Table 1].

## DISCUSSION

Cadmium exposure, produces toxicity by inducing ROS production through Fenton reaction,[26] inhibiting Ca^2+^ export from cytoplasm which in turn elevates cytoplasmic Ca^2+^ levels[27] and reduces the antioxidant defense systems,[28] there by producing oxidative stress. The mammalian cells possess elaborate defense mechanism for free radical detoxification. Nonenzyme molecules including thiols and disulfide bonding (balance between GSH-reduced glutathione and GSH-oxidized glutathione) play important roles among all antioxidant defense systems. The GSH donates electrons to the superoxide anion and hydroxyl radical, and hence prevents lipid peroxidation, DNA strand breakage, and oxidation of any organic molecules. Major reason for majority of toxicities has been reported to be the balance between the amount of free radicals generated in body and antioxidants to scavenge them and protect the body against their deleterious effects.[29]

In the present study, birds treated with cadmium group 2 showed significant higher values of erythrocyte SOD and CAT compared with the control group 1 at the end of fourth week, which indicates increased production of ROS by cadmium ions. After treatment with Indian medicinal plants from fourth to sixth week, the erythrocyte SOD and CAT values were reduced significantly.

In liver and kidney tissues, the concentration of GSH reduced in cadmium control group 2 compared with basal diet control group 1, which indicates the attachment of cadmium to SH groups of GSH and other proteins and persists in the tissues. Alteration of erythrocyte SOD and CAT and liver and kidney tissue GSH indicated disturbance of cellular antioxidants and increase in the production of oxygen free radicals which in turn causes lipid peroxidation that is indicated by increase in concentration of TBARS. Increase in LPO and other effects caused by oxygen free radicals produces damage to liver tissue which is revealed by increase in serum ALT enzyme concentration, which indicates inability of the liver to metabolize the ALT. Kidney damage was revealed by increase in BUN and serum creatinine concentration.

In the present study, *Withania somnifera* and *Ocimum sanctum* reduced the erythrocyte SOD and CAT activity and increased GSH and decreased TBARS of liver and kidney tissues, which reveals that the sparing of tissue GSH binds to cadmium ions and scavenges the ROS, thereby sparing the SOD and CAT. Once the LPO reduced, TABARS concentration was reduced and liver and kidney tissues were regenerated, as indicated by reversal of ALT and albumin for liver and creatinine and BUN for kidney. *Withania somnifera* and *Ocimum sanctum* are known for their antioxidant properties which are attributed to their antioxidant principles such as withanolides of *Withania somnifera*[30,31] and methyl eugenol and flovonoids of *Ocimum sanctum*.

In conclusion, the present study suggest that the herbal adaptogens *Withania somnifera* and *Ocimum sanctum* administration at the rate or 0.1% in feed significantly reversed the cadmium-induced oxidative damage and restored the liver and kidney functions and body weights.

## References

[1] Zhai L, Liao X, Chen T, Yan X, Xie H, Wu B (2008). Regional assessment of cadmium pollution in agricultural lands and the potential health risk related to intensive mining activities. A case study in Cnenzhou city, China. J Environ Sci (China).

[2] Liao XY, Chen TB, Xie H, Liu YR (2005). Soil as contamination and its risk assessment in areas near the industrial districts of chenzhou city, Southern china. Environ Int.

[3] Satarug S, Baker JR, Urbenjapol S, Haswell-Elkins M, Reilly PE, Williams DJ (2003). A global perspective on cadmium pollution and toxicity in non-occupationally exposed population. Toxicol Lett.

[4] Akyolcu MC, Ozcelik D, Dursun S, Toplan S, Kahraman R (2003). Accumulation of cadmium in tissue and its effect on live performance. J Phys IV France.

[5] Uyanik F, Even M, Atasever A, Tuncoku G, Kolsuz AH (2001). Changes in some Biochemical Parameters and organs of broilers exposed to cadmium and effect of zinc on cadmium induced alterations. Israel J Vet Med.

[6] Heyno E, Klose C, Krieger-Liszkay A (2005). Origin of cadmium-induced reactive oxygen species production: mitochondrial electron transfer versus plasma membrane NADPH oxidase. New Phytol.

[7] Quig D (1998). Cysteine metabolism and metal toxicity. Altern Med Rev.

[8] Bagchi D, Vuchetich PJ, Bagchi M, Hassoun EA, Tran MX, Tang L (1997). Induction of oxidative stress by chronic administration of sodium dichromate (chromium vi) and cadmium chloride (cadmium II) to rats. Free Radic Biol Med.

[9] Kara H, Karatas F, Canatan H, Serv K (2005). Effect of exogenous metallothionein on acute cadmium toxicity in rats. Biol Trace Elem Res.

[10] Stohs SJ, Bagchi D (1994). Oxidative mechanisms in the toxicity of metalions. Free Radic Biol Med.

[11] Kaczmarski M, Wojicicki J, Samochowiee L, Dutkiewicz T, Sych Z (1999). The influence of exogenous antioxidants and physical exercise on some parameters associated with production and removal of free radicals. Die Pharmazie.

[12] Kulkarni SK, Dhir A (2008). *Withania somnifera*: An Indian ginseng. Prog Neuropsychopharmacol Biol Psychiatry.

[13] Mishra LC, Singh BB, Dagenais S (2000). Scientific basis for the therapeutic use of *Withania somnifera* (Aswagandha): A review. Altern Med Rev.

[14] Sankar SR, Manivasagam T, Krishnamurt A, Ramanathan M (2007). The neuroprotective effect of Withania somnifera root extract in MPTP-intoxicated mice: An analysis of behavioral and biochemical variables. Cell Mol Biol Lett.

[15] Prakash P, Gupta N (2005). Therapeutic uses of *Ocimum sanctum Linn (Tulsi)* with a note on eugenol and its pharmacological actions: a short review. Indian J Physiol Pharmacol.

[16] Monica B, Gupta RY (2004). Effect of *Ocimum sanctum* (Tulsi) leaf powder on clinicopathological and immune response in chicken. Hariyana Vet.

[17] Panossian AG (2003). Adaptogens: Toxic herbs for fatigue and stress. Altern Ther Health Med.

[18] Calliborne AL, Greenwald RA (1985). Assay of catalase Hand book of oxygen Radical Research.

[19] Markulund S, Markulund G (1974). Involvement of the superoxide anion radical in the autooxidation of pyrogallol and a convenient assay for superoxide dismutase. J Comp Physiol B.

[20] Moron MS, Depierre JW, Mannervik B (1979). Levels of glutathione, glutathione reductase and glutathione S transferase in rat lung and liver. Biochem Biophys Acta.

[21] Subramanian KA, Manohar M, Mathan VI (1988). An unidentified inhibitor of lipid peroxidation in intestinal mucosa. Biochim Biophys Acta.

[22] Bergmeyer HU, Horder M, Rej R (1986). Approved recommendation (1986) Approved recommendation (1985) on IFCC methods for the measurement of catalytical concentration of enzyme, part 3. IFCC for alanine aminotransferase. J Clin Chem Clin Biochem.

[23] Apple F, Bandt C, Prosch A, Erlandson G, Holmstrom V, Scholen J (1986). Creatinine clearance: enzymatic vs Jaffe determinations of creatinine in plasma and urine. Clin Chem.

[24] Wybenga DR, Di Giorgio J, Pileggi VJ (1971). Manual and automated methods for urea nitrogen measurement in whole serum. Clin Chem.

[25] Lowry OH, Rosebrough NJ, Farr AL, Randall RJ (1951). Protein measurement with the Folin phenol reagent. J Biol Chem.

[26] Liochev SI, Sigel A, Sigel H (1999). The mechanism of “Fenton-like reactions and their importance for biological systems. A biologists’ view. In Metal Ions in Biological Systems.

[27] Goyer RA, Clarkson TW (2001). Toxic effects of metals. Casarett and Doull’s Toxicology. The basic Science of Poisoning.

[28] Wim W, Detmar B (2004). Cadmium induced apaptosis in C6 glioma cells: Influence of oxidative stress. Biometals.

[29] Sies H (1993). Strategies of antioxidant defense Eur J Biochem.

[30] Davis L, Kuttan G, Kuttan G (2001). Effect of *Withania somnifera* on dibenzyl benzanthracene (DMBA) induced carcinogenesis. J Ethanopharmacol.

[31] Prakash J, Gupta SK, Dinda AK (2002). *Withania somnifera* root extract prevents DMBA induced squamous cell carcinoma of skin in swiss albino mice. Nutr Cancer.

